# The cartilage matrisome in adolescent idiopathic scoliosis

**DOI:** 10.1038/s41413-020-0089-0

**Published:** 2020-03-09

**Authors:** Carol A. Wise, Diane Sepich, Aki Ushiki, Anas M. Khanshour, Yared H. Kidane, Nadja Makki, Christina A. Gurnett, Ryan S. Gray, Jonathan J. Rios, Nadav Ahituv, Lila Solnica-Krezel

**Affiliations:** 1 Center for Pediatric Bone Biology and Translational Research, Texas Scottish Rite Hospital for Children, 2222 Welborn St., Dallas, TX 75219 USA; 2 McDermott Center for Human Growth and Development, University of Texas Southwestern Medical Center, Dallas, TX 75235 USA; 3 Departments of Pediatrics, University of Texas Southwestern Medical Center, Dallas, TX 75235 USA; 4 Orthopaedic Surgery, University of Texas Southwestern Medical Center, Dallas, TX 75235 USA; 5 Department of Developmental Biology, Washington University School of Medicine, St. Louis, MO 63110 USA; 6 Department of Bioengineering and Therapeutic Sciences, University of California San Francisco, San Francisco, CA 94158 USA; 7 Institute for Human Genetics, University of California San Francisco, San Francisco, CA 94158 USA; 8 Department of Anatomy and Cell Biology, University of Florida, College of Medicine, Gainesville, FL 32610 USA; 9 Departments of Neurology, Washington University School of Medicine, St Louis, MO 63110 USA; 10 Pediatrics, Washington University School of Medicine, St Louis, MO 63110 USA; 11 Orthopaedic Surgery, Washington University School of Medicine, St Louis, MO 63110 USA; 12 Department of Pediatrics, Dell Pediatric Research Institute, University of Texas at Austin Dell Medical School, Austin, TX 78723 USA

**Keywords:** Pathogenesis, Bone quality and biomechanics

## Abstract

The human spinal column is a dynamic, segmented, bony, and cartilaginous structure that protects the neurologic system and simultaneously provides balance and flexibility. Children with developmental disorders that affect the patterning or shape of the spine can be at risk of neurologic and other physiologic dysfunctions. The most common developmental disorder of the spine is scoliosis, a lateral deformity in the shape of the spinal column. Scoliosis may be part of the clinical spectrum that is observed in many developmental disorders, but typically presents as an isolated symptom in otherwise healthy adolescent children. Adolescent idiopathic scoliosis (AIS) has defied understanding in part due to its genetic complexity. Breakthroughs have come from recent genome-wide association studies (GWAS) and next generation sequencing (NGS) of human AIS cohorts, as well as investigations of animal models. These studies have identified genetic associations with determinants of cartilage biogenesis and development of the intervertebral disc (IVD). Current evidence suggests that a fraction of AIS cases may arise from variation in factors involved in the structural integrity and homeostasis of the cartilaginous extracellular matrix (ECM). Here, we review the development of the spine and spinal cartilages, the composition of the cartilage ECM, the so-called “matrisome” and its functions, and the players involved in the genetic architecture of AIS. We also propose a molecular model by which the cartilage matrisome of the IVD contributes to AIS susceptibility.

## Introduction

**Fig. 1:**
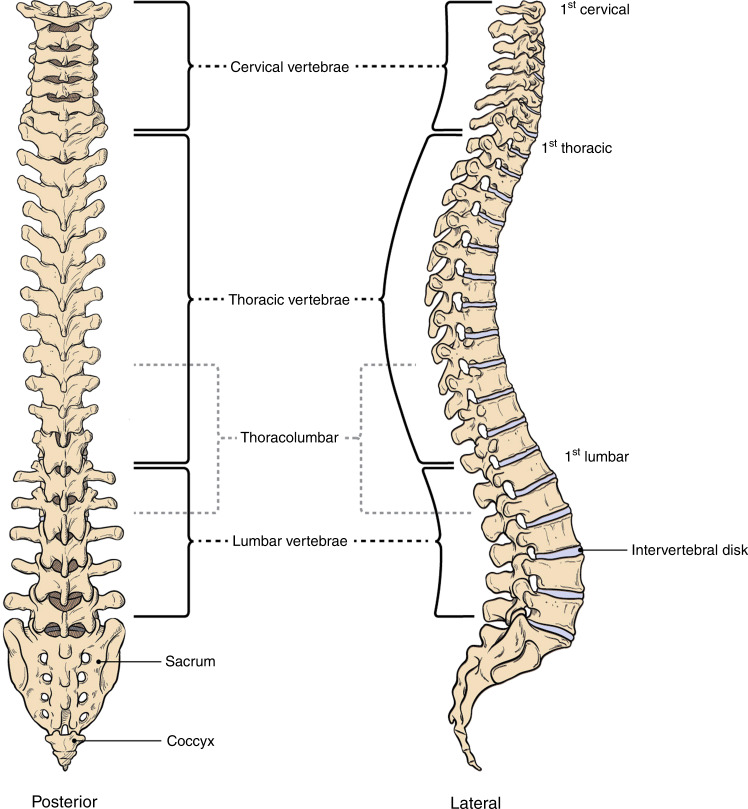
The human spine viewed from the back (posterior) and side (lateral)

**Fig. 2:**
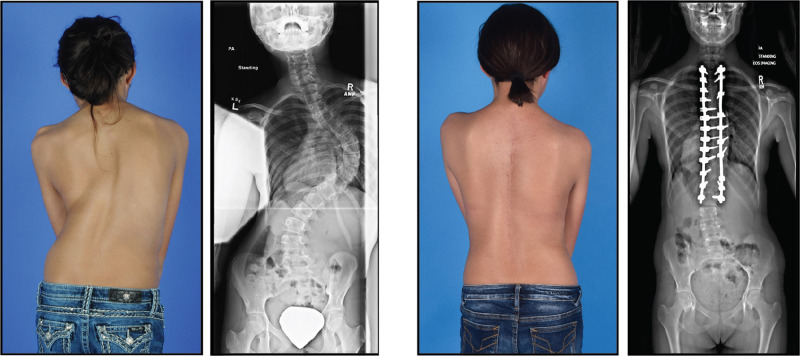
Adolescent idiopathic scoliosis in a female patient. Photograph and standing radiograph (left) reveal a typical right-sided thoracic curve and postural imbalance that is largely corrected with surgical implants (right)

The human spine serves multiple structural and physiologic roles. An essential function of the spinal column in all vertebrates is to integrate the brain and nervous system with the axial skeleton and simultaneously protect the spinal cord. For humans, the symmetry and position of the spine is key for bipedalism and for maintaining the center of gravity over the pelvis.^1^ The spine, with its 33 vertebrae, 23 intervertebral discs (IVDs), ligaments, and facet joints, withstands biomechanical forces while providing flexibility in three dimensions (Fig. 1). The stability of this multicomponent system over a lifetime is remarkable, although eventually the wear and tear from environmental stresses, plus genetic contributions, can evoke degenerative changes primarily in the IVD of the lumbar spine.^2^ Other common pathologies of the adult spine include tumors, fractures, lumbar spondylosis, stenosis, and segmental instability or deformity. Scoliosis is a lateral spinal deformity that is common and exhibits increasing prevalence with age and degeneration of the spine.^3^ In children, rare developmental disorders of embryogenesis due to malformed vertebrae, muscle weakness, or neurologic impairment can affect the integrity of the spine.^1^ However, the most frequent spinal disorder presenting in children is idiopathic scoliosis, which unlike adult scoliosis occurs in the absence of other associated pathologies or degenerative changes. Idiopathic scoliosis, defined by lateral deformity greater than 10 degrees on coronal radiographs, usually presents during the adolescent growth spurt. Adolescent idiopathic scoliosis (AIS) is the most common pediatric musculoskeletal disorder, occurring in ~3% of school age children, >29 million children worldwide^4^ (Fig. 2). Untreated, progressive AIS is associated with restrictive lung disease, pain, and severe deformity later in life.^5^ Although curve patterns vary, right-sided thoracic deformities are typical for reasons that are not understood. A child whose scoliosis continues to worsen before the peak of growth velocity is at greatest risk of serious deformity, but after skeletal maturity the risk of “curve progression” decreases sharply.^6^ Orthotics, i.e., a back bracing, may be prescribed to help control progression until skeletal maturity, and not to necessarily correct existing deformity.^7,8^ Bracing is considered to have “failed” if the curve continues to progress to ~50 degrees before closure of growth plates as measured from iliac crest or hand X-rays.^9^ In this scenario, the standard of care is surgical correction, with the goal of reducing the deformity, fusing the involved vertebral bodies to prevent continued progression, and achieving balance of the head, shoulders, and trunk over the pelvis. Typical surgery involves implanting metal instrumentation to provide the forces to straighten and derotate the deformity.^1^

For centuries, physicians have speculated on the causes of AIS.^10^ In what tissues does it originate, and why the curious onset during the adolescent growth spurt? Case reports and epidemiologic studies dating back more than 45 years have consistently demonstrated significant but complex heritability in AIS.^11–13^ The assumption from these models is that AIS, in a typical patient, arises from a cumulative effect of several to many genetic risk factors. Certain patients, however, may harbor more deleterious genetic factors, for example, those with a family history of the disease, that increase their risk of developing a scoliosis phenotype. Another curious feature of AIS is its striking sexual dimorphism, with girls having a more than fivefold greater risk than boys of progressive deformity requiring treatment.^14^ These observations suggest that the genetic factors underlying AIS may differ between the sexes. In this model, it is hypothesized that the total mutational burden required to “activate” progressive AIS in males will be greater than that in females, a phenomenon known as the Carter effect.^15,16^ It follows from this concept that males with progressive AIS will harbor more high-risk, deleterious mutations. As with other complex genetic disorders, we expect many heritable mutations with varying effect sizes and frequencies will comprise the total mutational burden in AIS. Variants that are rare in a population tend to be more penetrant and confer a greater disease risk in an individual, whereas common variants are expected to be less penetrant and to confer relatively smaller effects.^17^

The underlying genetic, molecular, and cellular causes of AIS have remained enigmatic for centuries. However, modern genomic and systems biology approaches in human patient cohorts are beginning to elucidate the genetic changes underlying AIS risk. Simultaneously, precise and powerful forward and reverse genetic approaches in vertebrate model systems are enabling researchers to rapidly identify new AIS candidate genes that may be screened in clinical cohorts, as well as to directly test the phenotypic and mechanistic consequences of mutations discovered in human patients. From these efforts the field of AIS research has seen a convergence on factors that control the development of the IVD, particularly on molecules that maintain or comprise the extracellular matrix (ECM). For the rest of this review, we focus on the biologic factors that control IVD development and their genetic contributions to AIS susceptibility. We also discuss the implications of these discoveries for new hypothesis-driven research and therapeutic interventions.

## Biologic pathways of spine development

**Fig. 3:**
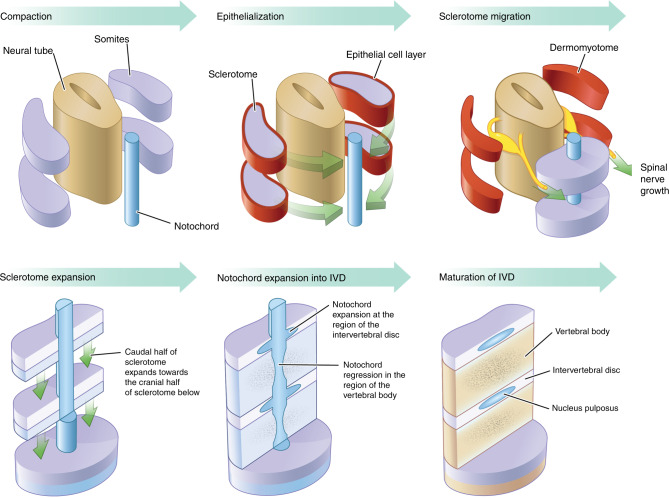
Cartoon depiction of the stages of somitogenesis and maturation of the intervertebral disc. Somites (lavender) become epithelialized to form the dermomyotome (red) and sclerotome. Cells of the sclerotome migrate from pairs of paraxial somites and condense around the notochord in a characteristic pattern of more or less condensed regions. These alternating regions (blue and lavender in the figure) give rise to the vertebral bodies and AF, respectively. The NP arises by a different developmental path by swelling in the region of the IVD and pinching off of the notochord in the vertebral bodies

The diverse functions of the spine in strength, flexibility, balance, and protection are enabled by a striking segmented organization along the vertical axis that is the product of an exquisitely controlled molecular network called the “segmentation clock”. The details of the segmentation clock have been described in a number of excellent reviews to which we refer the reader.^18,19^ Briefly, in early development, presomitic mesodermal cells proliferate and move from the tail bud at a specific rhythmic pace, creating somites that emerge at the anterior tip and progress along the embryonic axis (Fig. 3). The somites then develop into a dorsal epithelial dermomyotome that differentiates into skin and muscle, and a ventral sclerotome that forms the vertebral bodies, ribs, tendons, and cartilages of the spine, i.e., the vertebral growth plates and IVDs. In humans, a pair of somites forms every 6–12 h between days 20 and 32 of embryonic development.^20^ This finely timed process is controlled by three major signaling pathways—Fibroblast Growth Factor (FGF), Notch, and Wnt/β-catenin—that set up waves of gene expression through “circuits” controlling the timing of oscillations, spatial expression, and synchronicity of somitogenesis.^18,21^ The subsequent formation of the sclerotome, i.e., future spine and associated structures, requires that a subset of mesodermal cells in the ventral somite region undergo an epithelial-to-mesenchymal transition and then migrate toward the midline to surround the notochord (axial mesoderm). This two-step transition is regulated by Wnt and Fgf signaling pathways.^22^ The vertebrate spine also derives from the axial mesoderm in addition to the paraxial mesoderm (somites) (Fig. 3).

## Developmental regulation of IVD biogenesis

In early mouse embryonic development, the sclerotome migrates out from the somites to surround the notochord in a pattern of condensed and less condensed segments corresponding to future IVD and vertebral bodies, respectively, along the anterior/posterior axis. The paired box 1 (Pax1) transcription factor is a well-described marker of sclerotome development which is induced via factors secreted from the notochord including: Sonic Hedgehog (Shh) and through noggin-dependent inhibition of bone morphogenetic protein (BMP) signaling.^23^ During embryonic spine development, the notochord regresses from regions of developing vertebral bodies and expands in regions of the future IVD (Fig. 3). The mechanism of this physical transformation of the notochord remains unresolved but may be related to the mechanical force exerted by the developing vertebral bodies.^23–25^ Eventually, the notochord tissue gives rise to the nucleus pulposus (NP), the gel-like inner core of the IVD, and *Pax1* expressing cells contribute to the region of the outer annulus fibrosus (AF) IVD.^26^ The spontaneous mutant mouse strains “*undulated*” and “*scoli*” display varying spinal phenotypes resulting from mutations in the *Pax1* locus.^27–30^ Two undulated strains (*un*, *Un*^*ex*^) exhibit mild kink tail phenotypes due to hypomorphic *Pax1* mutations. By comparison, the homozygous *Un*^*s*^*/Un*^*s*^ strain harbors a 120 kb deletion encompassing *Pax1* and completely lacks lower thoracic and lumbar vertebral units. Of note, *Un*^*s*^*/Un*^*s*^ displays a more severe phenotype than a homozygous targeted knockout of *Pax1*, suggesting that noncoding sequences outside of *Pax1* may drive expression of additional loci related to spine development. Indeed, analysis of *Pax1* enhancers using transgenic assays in mouse identified a strong extra-genic enhancer “Xe1” which drives expression in the sclerotome and whose loss presumably contributes to the *Un*^*s*^*/Un*^*s*^ phenotype.^31^ More comprehensive analysis of enhancers, using transcriptomic and chromatin immunoprecipitation sequencing (ChIP-seq) studies of Pax1-positive flow-sorted E12.5 vertebral column cells, found enrichment of signals at loci related to ECM, cartilage development, and collagen fibrillogenesis biological pathways. Moreover, Pax1-negative cells display a general downregulation of ECM genes, e.g., *Col11A1*, suggesting Pax1 is a critical transcriptional regulator of ECM matrix biogenesis in the outer AF. Proteoglycan accumulation (e.g., aggrecan) is also significantly reduced in animals lacking *Pax1*.^32,33^ It is clear from these and other studies that *Pax1*, together with its paralog *Pax9*, act synergistically to drive development of IVD of the entire vertebral spine.^34^

**Fig. 4:**
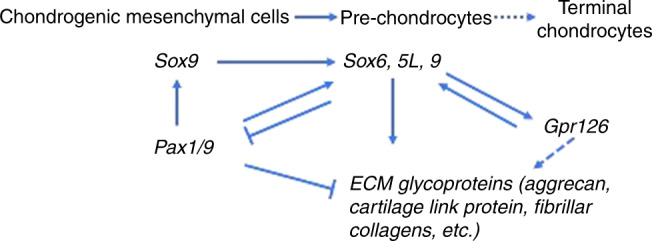
Schematic of the *Pax/Sox*-mediated extracellular matrix pathway in cartilages and IVD. Genes that have been associated with AIS are shown in bold. *Pax1* expression precedes expression of the *Sox* trio in IVD development. The *Sox* trio directly activates the transcription of many *ECM* genes. *Gpr126* and *Sox9* positively regulate each other in IVD and chondrocyte cells. In the presence of *Sox9*, *Pax1/9* may inhibit expression of aggrecan and possibly other ECM genes in a context-dependent manner

Sox9 is a master transcriptional regulator of chondrogenesis.^33,35–38^ *Sox5/6* is expressed in the notochord and the sclerotome, where they share partially redundant roles and cooperate with Sox9 to drive the expression of chondrogenesis genes in the growth plate.^39–44^ Sox5 and 6 are clearly important in IVD development as well. Both are expressed in the notochord and the sclerotome, and the notochordal sheath fails to form in *Sox5*^−/−^;*Sox6*^−/−^ mice. The consequence of this is failure of the NP to form. In addition to severe chondrodysplasia, mice lacking *Sox6* alone develop a kinked tail that occurs in segments where the NP is small and misplaced,^43,45^ while *Sox5*^−/−^*;Sox6*^−/−^ double mutant mice display a virtual absence of cartilage and chondrogenic tissues of the spine.^46^ Other striking features of *Sox5*^−/−^*;Sox6*^*−/*−^ double mutant mice are the downregulation of genes encoding major ECM components including collagen II, aggrecan, and cartilage link protein, and a general delay in differentiation of the inner AF (IAF).^32,45,47^ Sox9 controls *Sox5/6* expression and is required for the normal patterning and development of the spine, failing to generate obvious patterned vertebral bodies or IVD tissues when ablated in embryonic mice.^48^ Sox9 is continuously required for homeostasis of the spine in adult mice, where its ablation in skeletally mature mice leads to kyphoscoliosis, disc compression, and degeneration of the IVD.^37^ Interestingly, *Pax1* transcripts are upregulated when *Sox5* and *6* together are removed genetically from the IVD anlagen, suggesting regulatory feedback between Sox5/6 and Pax1 signaling, which may help to define the regulatory programs driving development of the inner and outer AF. Indeed, Pax1, as well as its paralog Pax9, also assists in the robust gene expression of *Sox5*. Thus, Pax1/9 and Sox9 function in concert with BMP and transforming growth factor (TGF) signaling and are critical to initiate expression of chondrogenic genes during early IVD differentiation. Later, the expression of *Pax1* is restricted to the outer annulus by the *Sox5/Sox6* negative feedback mechanism^26,49^ (Fig. 4).

## Bearing the load—the postnatal intervertebral disc

**Fig. 5:**
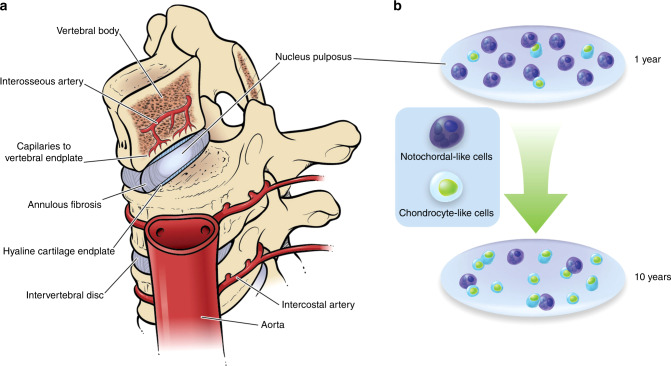
The human IVD. The IVD is largely avascular, receiving nutrition through the cartilaginous endplate. Callout depicts replacement/conversion of notochordal-like cells in the nucleus pulposus with chondrocyte-like cells in the first 10 years of life

In humans, the spine grows most rapidly in the first 5 years of life, with an average T1 to S1 segment length increase of 10 cm during this time (2 cm per year). There is a slower T1 to S1 growth from age 5 to 10 years of ~5 cm until adolescence (1 cm per year). From age 10 years to adulthood, T1 to S1 grows an additional 10 cm; this includes the adolescent growth spurt (2 cm per year).^50^ There are 23 IVDs in the fully formed human spine that collectively contribute about one third of its height.^51^ The vertebrate IVD is composed of fibrocartilaginous tissues encapsulating a gel-like NP tissue, and consequently is considered a cartilaginous tissue joint that connects the vertebral bodies (Fig. 5a). Cells within the IVD are described as “chondrocyte-like” and “fibroblast-like”. Within the IVD, the NP is surrounded by a lamellar structure aptly named the AF. The ring-shaped AF has two distinct parts, an inner AF made of chondrocytic cells nearest the NP and expressing type II collagen, and an outer AF made of fibroblast-like cells expressing mostly type I collagen. In the outer AF, concentric lamellae of collagen fibers are organized in alternating angles that are oblique to the spinal axis, a so-called “angle-ply” structure. This arrangement conveys mobility and flexibility in multiple planes of motion.^23,52^ The IVD is capped by the cartilage endplate (CEP) that connects to cartilage fibers extending from the AF.

The CEP has the critical role of supplying oxygen and nutrients to cells within the IVD, which is in fact the largest avascular structure in the body (Fig. 5a). While the cells of the outer AF are fed by capillaries in the surrounding soft tissues, the remaining cells of the AF and NP receive their nutrients from capillaries that penetrate the vertebral bodies and terminate in loops within the CEP. Nutrients and metabolites then move to and from cells of the disc through the CEP and dense ECM, mostly by diffusion. The IVD is relatively acellular, with about 9 000 “fibroblast-like” cells per mm^3^ in the AF and only 4 000 “chondrocyte-like” cells per mm^3^ in the NP.^52^ In the latter case, notochordal-like cells that begin as large vacuolated structures are slowly replaced by distinctly smaller and less metabolic cells in postnatal development, up to ~10 years of age in humans (Fig. 5b). At this point, notochordal-like cells are virtually absent and replaced by cells with a chondrocyte-like morphology.^23^ The transition of cells to a chondrocyte-like phenotype coincides with a change in ECM composition and firming of the NP due to collagen fibril accumulation. The precise transition states of the cells within the NP, and underlying mechanism driving their phenotypes, are still unclear. Cell-tracing experiments using specific Cre drivers such as *Shh-Cre* and *Noto-Cre* demonstrate that cells of notochordal origin are detected in adult NP cells of the mouse.^24^ However, not all cells are labeled in these experiments, pointing to heterogeneity that could be due to spatio-temporal differences in NP cell content or even differing origins. Clearly a key role of the NP is to orchestrate the production of healthy ECM.^53^ Experimental evidence suggests that the large notochordal-like “precursor” cells produce proteoglycans faster than small chondrocyte-like cells. Moreover, the distribution and assembly of proteoglycans differs between the two cell types.^54^ Consequently, it is reasonable to conjecture that the proportions of cell types within the NP may determine ECM production and thereby influence the strength and integrity of the IVD.

## Composition and role of the intervertebral disc matrisome

**Fig. 6:**
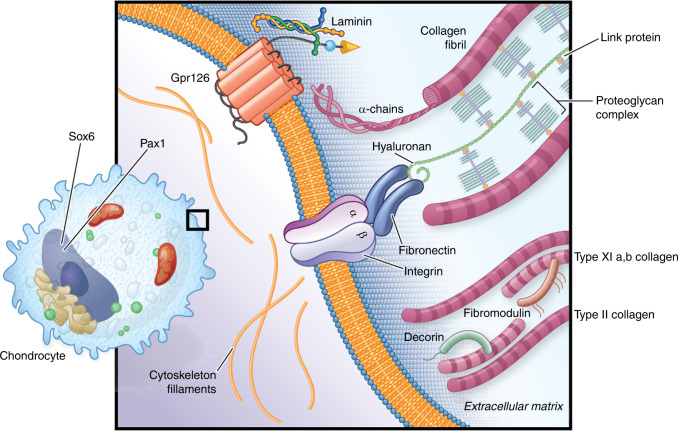
Cartoon depiction of a cartilage cell and surrounding matrix. Fibrillar collagens are shown in pink, while sulfated proteoglycan (e.g. aggrecan, perlecan) is shown as a complex with link protein and hyaluronic acid. The adhesion G protein receptor GPR126 is depicted interacting with a laminin ligand, although its physiologic interaction in cartilage has not been described

In the intact disc, osmotic swelling within the NP is resisted radially by the collagen fibers of the AF and axially by the endplates and vertebrae. The AF then effectively absorbs compressive and torsional loads.^23,52^ Thus, the three components of the disc are “greater than the sum of the parts”, serving to support and maintain each other as they collectively bear loading of the spine while conferring mobility. Deficiencies in any one part of the disc can impact the whole and, in the mature organ, lead to irreversible damage. The ECM is a critical functional compartment of the IVD, providing mechanical strength, regulating nutritional flow as noted, and conveying physiologic signals back to the cells^55^ (Fig. 6). The composition of the acellular ECM, the so-called “matrisome” has been described in a number of excellent reviews to which we direct the reader.^56–58^ Here we describe the ECM in brief, with particular reference to the cartilaginous matrisome of the IVD. Predominant components of the cartilage matrisome are fibrillar collagens, chondroitin sulfate proteoglycans, and hyaluronic acid. These large molecules form the basic structural network of the matrisome that allows other factors and cells to interact.^56,58^ The IVD produces multiple collagens including types I, II, III, V, VI, IX, XI, XII, and XIV. Types I and II collagens are predominant in the AF and NP, respectively as noted. The minor, less abundant collagen types V and XI form hybrid fibrils with types I and II collagens, respectively, where they are thought to help maintain an appropriately narrow diameter to the collagen fibrils in each compartment. Proteomic studies of the bovine NP suggest that various heterotrimeric combinations may be represented in the type V/XI collagen pool of the disc. What determines this composition is not clear, but may devolve from preferences of the different C-propeptide domains of the various procollagen chains, their relative expression levels in the endoplasmic reticulum where the helices are formed, and/or differences in cell phenotype within the NP both spatially and temporally, for example chondrocytes versus notochordal cells.^59^ While collagen is the primary structural component of the disc ECM, elastin fibers are also key for structural support and resistance to deformation.^51,57^

The proteoglycan content is highest in the NP and declines outward toward the AF. In the NP, chondroitin sulfate-linked proteoglycans interact with collagens to retain hydration and form a gel-like matrix that resists compression and provides height to the disc. The predominant proteoglycan of the IVD matrisome is aggrecan, present in both NP and AF.^23^ Aggrecan is aptly named as it aggregates along chains of hyaluronan, a high molecular weight polysaccharide, through a small “link” protein called cartilage link protein.^60^ The overall negative charge of this complex attracts water and leads to osmotic swelling within the loose meshwork of collagen fibrils in the NP^23,51,61^ (Fig. 6). Aggrecan is composed of a core protein linked to both chondroitin sulfate and keratan sulfate chains. Chondroitin sulfate is the major side chain at birth, but over time the proportion of keratan sulfate substitution increases. The shift in side chain composition of aggrecan seems to mirror the transition of notochordal-like cells to chondrocyte-like cells and may reflect differing aggrecan production by the two cell types and less access to oxygen that is needed for chondroitin sulfation intermediates.^52,53^ Hyaluronan and link protein also change in abundance over time in the disc. While hyaluronan increases, link protein abundance decreases with age and its proteolytic processing increases. There is evidence that a peptide called “N-link” released by this process actually stimulates ECM (glycosaminoglycan and collagen) production via BMP2 receptor SMAD1/5 signaling.^62,63^

Other proteoglycans in the disc function as accessory proteins that may, for example, participate in collagen assembly and help to regulate the appropriate width and length of fibrils. For example, small leucine-rich repeat proteins (SLRPs) are proteoglycans such as versican, decorin, biglycan, fibromodulin, lumican, and perlecan that contain characteristic domains including carboxy-terminal leucine-rich repeats (Fig. 6). These proteins regulate collagen fibril growth rates, size, content, and width.^56^ Other proteins effectively bridge the structural components of the ECM, re-enforcing and strengthening the network and also connecting it to cells and to other soluble factors. The multidomain structures of these connectors facilitate direct binding to cell surface receptors, other ECM connectors, and soluble growth factors. Example glycoproteins in this class include the fibronectins and laminins. Like collagens, laminins are triple-helical proteins made up of α, β, and γ chains that combine via a central coiled-coil domain. Multifunctional domains of the laminins can interact with cell surface receptors such as integrins and also other components of the ECM, thereby mediating cell–cell interactions. Fibronectin, a large secreted glycoprotein, dimerizes through C-terminal disulfide bonds where it binds to collagens, gelatins, integrins, and other ECM molecules. In particular, fibronectin binding to integrin receptors promotes their clustering in the cell membrane, which in turn leads to further assembly of fibronectin into fibrils around the cells.^56–58^ In the adult disc, fibronectin proteolysis into fragments is correlated with the grade of disc degeneration, and has been shown to stimulate catabolic events that lead to the same.^52^

## Organization of the IVD matrisome

Within the disc, the AF is a collagen-rich fibrillar ECM constructed to withstand tensile and torsional forces transmitted outward from the gelatinous NP. Microfibrils are an important structure distinct from collagen and elastin fibrils.^51,57^ The primary structural component of microfibrils in the IVD are the large, structurally distinctive fibrillin glycoproteins (FBN1, 2, 3). Associated with the fibrillins are a number of ancillary proteins, such as microfibril-associated glycoprotein 1, that provide specialized functions. In the OAF and IAF microfibrils co-localize with elastin fibers. While in the OAF microfibrils closely align with collagen lamellae of the ECM, in the IAF fibrillin-positive microfibrils are also seen in an elastin-negative region around cells. In the NP microfibrils are found almost exclusively in the pericellular region, away from elastin fibers in the ECM.^51^ The distribution of microfibrils may reflect differing roles in the OAF, which must absorb the brunt of compression and torsional forces, compared with the IAF and NP that are subjected to relatively minor, isotropic forces. It is also now appreciated that the microfibrillar niche is a command center for regulating cellular behavior. This is apparently achieved through direct interactions with cell surface receptors as well as by serving as a reservoir for various growth factors, particularly in the TGF family.^51,64,65^

## Pathways of IVD development and susceptibility to adolescent idiopathic scoliosis

The majority of genetic risk in AIS has been defined thus far by hypothesis-free genome-wide association studies (GWAS) that map the location of genetic risk factors in the genome relative to fixed markers, usually single nucleotide polymorphisms (SNPs).^66^ By definition, SNPs are common in the population and consequently can be informative in statistical tests such as logistic regression that measure frequency differences between affected cases and matched controls. Because of the many tests performed in a typical GWAS (so-called “multiple testing”), a significance level of *P* < 5 ×10^−8^ is generally required for any association to be declared significant (GWAS-level significance). Replication of GWAS-significant loci is necessary to independently validate candidate SNPs and provide realistic estimates of effect size and fraction of risk conferred across populations. It is important to note that while GWAS are proven to be powerful for mapping disease loci, further fine-mapping approaches are needed to define the underlying causal mutations. Multiple AIS risk loci that were originally identified in GWAS have been further validated by replication and meta-analysis studies, as given in Table 1. Importantly, while these studies have mapped disease loci near or within likely candidate genes, the majority have not pinpointed causal sequence changes driving genetic associations. Nonetheless, as shown below, the number of validated loci around genes that are known to determine early spine development is striking. Further, pathway enrichment analysis of a recent multiethnic GWAS meta-analysis provided statistically significant evidence that biologic processes of cartilage development and/or maintenance are impacted in AIS.^67^

**Table 1:** Susceptibility loci in AIS. Table of significant associations and OMIM-related phenotypes

Genome region	Marker	Candidate gene(s)	OMIM disorder	Reference
10q24.31	rs11190870	*LBX1*		^59^
6q24.1	rs6570507	*GPR126*	Lethal congenital contracture syndrome	^61^
9p22.2	rs3904778	*BNC2*		^76^
20p11.22	rs6137473	*PAX1*	Otofacial cervical syndrome 2	^42^
9q34.2	rs687621	*ABO*		^68^
11p15.2	rs1455114	*SOX6*	Craniofacial dyssynostosis with short stature	^68^
16q23.3	rs4513093	*CDH13*		^68^
18q21.33	rs4940576	*BCL2*		^62^
2q36.1	rs13398147	*PAX3/EPHA4*	Craniofacial-deafness-hand syndrome	^62^
1p36.32	rs241215	*AJAP1*		^62^
17q24.3	rs12946942	*SOX9, KCNJ2*	Campomelic dysplasia/familial atrial fibrillation	^60^
11q24.1	rs35333564	*MIR4300HG*		^68^
4q21.23	rs141903557	*LOC101928978*		^111^
1q21.2	rs11205303	*MTMR11*		^96^
1q42.13	rs12029076	*ARF1*		^96^
22q11.21	rs1978060	*TBX1*	Velocardiofacial syndrome	^96^
12p12.3	rs2467146	*LINC02378/MIR3974*		^96^
8p23.2	rs11787412	*CSMD1*		^96^
9p13.3	rs188915802	*KIF24*		^96^
6q14.1	rs658839	*BCKDHB/FAM46A*	Osteogenesis imperfecta type XVIII	^96^
7p15.1	rs160335	*CREB5*		^96^
6q22.1	rs482012	*NT5DC1*		^96^
7p22.3	rs11341092	*LOC101927021/UNCX*		^96^
1q32.2	rs17011903	*PLXNA2*	Beckwith–Wiedemann syndrome	^96^
7p21.2	rs397948882	*AGMO/MEOX2*		^96^
16q12.2	rs12149832	*FTO*	Growth retardation, developmental delay, and facial dysmorphism	^96^

Regions in the human genome providing statistically significant evidence of harboring an AIS risk factor are denoted by their OMIM locus number (column one) where applicable. The markers shown are those that provided most significant evidence in the region

An association distal to the *PAX1* gene was originally discovered by a GWAS in 3 102 individuals of European descent (North American) and replicated in independent North American, Japanese, and East Asian female cohorts.^68^ Further investigation revealed that the association was driven almost entirely by females (combined rs6137473 *P* = 2.15 × 10^−10^, OR = 1.30) but not males (combined rs6137473 *P* = 0.71, OR = 1.08), identifying it as an apparently sex-specific AIS locus. Resequencing highly conserved sequence elements within the associated interval in AIS subjects refined the signal to a putative enhancer near the *Xe1* enhancer described earlier. The new enhancer, called *PEC7*, was shown to drive reporter gene expression in zebrafish somites, an activity that was disrupted by AIS-associated SNPs.^68^ These data suggest that alterations in the spatial and/or temporal control of *PAX1* expression can increase risk of spinal deformity (*AIS*). The association signal within an intron of the *SOX6* gene implicates its transcriptional regulation in AIS. *SOX6* is expressed in several tissues including testis, muscle, and spinal cord in addition to chondrocytes.^45^ However, it is tempting to speculate that AIS-associated variation in the *SOX6* enhancer could specifically diminish its expression in cartilaginous tissues and thus dysregulate *SOX5/6/9*, *PAX1*, and possibly even *GPR126* (see below) in these tissues. *GPR126* (OMIM 612243, alias *ADGRG6*, or *VIGR*) is a member of the adhesion G protein-coupled receptor family with multiple cellular functions ranging from cell adhesion to migration, to name a few.^69^ In humans, *GPR126* is highly expressed in cartilages and the IVD. In mouse embryo, *Gpr126* is highly expressed in the proliferating cartilage of the spine, suggesting its role in spinal development could be exerted through effects on chondrogenesis.^70^ As outlined below, further experiments in conditional mutants have supported this hypothesis, and in particular, have shown that Gpr126 is a positive regulator of cartilage differentiation and possibly its homeostasis. In the chondrogenic ATDC5 cell line, *Gpr126* mRNA expression increases with early chondrogenic differentiation.^71^ *Gpr126* overexpression in this cell line increases expression of the cartilage marker genes *Col2a1* (encoding type II collagen) and *Acan* (encoding aggrecan), while *Gpr126* knockdown decreases their expression.^72^ Gpr126 and Sox9 are also positive regulators of each other, as knockdown or knockout of either reduces expression of the other in ATDC5 cells, chondrocytic N1511 cells, or adult IVD.^37,72^ In zebrafish, Gpr126 is required for Schwann cell myelination, an effect that could be overcome by elevating cAMP levels with forskolin, suggesting a classic signaling mechanism through G proteins.^73^ Gpr126 receptor signaling can be self-activated through autoproteolytic processing.^74^ Gpr126 additionally mediates certain activities such as Schwann cell development through interaction with a laminin-211 ligand.^75^ However, the mechanisms by which Gpr126 signals in chondrocyte cells are yet to be characterized.

Whether genetic variation of other associated loci contributes to mechanisms of cartilage biogenesis awaits investigation. For example, *CDH13* encodes the cadherin 13, or “T-cadherin” protein, a unique member of the cadherin family that lacks transmembrane and cytoplasmic domains, attaching to the plasma membrane via a glycosylphosphatidylinositol anchor.^76^ During development T-cadherin is highly expressed throughout the early developing sclerotome, with later expression restricted to the caudal sclerotome and migrating neural crest cells.^77^ Strong *CDH13* expression is also reported in a chondrocytic cell line and rib cartilage, but its role in these tissues is unknown.^67^ ABO encodes glycosyltransferase isoforms that catalyze the transfer of carbohydrates to the H antigen, forming the antigenic structures of the ABO blood group [MIM:616093]. *ABO* is expressed at varying levels in a variety of tissues and is reportedly undetected in cartilage. *BNC2* (OMIM 608669) encoding Basonuclin 2, is a highly conserved transcription factor belonging to the *C2H2* group of zinc finger proteins. In humans, BNC2 is produced in a variety of tissues including bone and cartilage. The strongest and most reproduced association with AIS in multiple studies is with SNP rs11190870 that is located near the end of the *LBX1* gene. *LBX1* is encoded head-to-head with another gene *FLJ41350* (also called *LOC399806* or *LBX1-AS1* (for *LBX1* antisense RNA 1)). *LBX1* (OMIM 604255) encodes the ladybird late homeobox transcription factor. *Lbx1* is specifically expressed early in embryogenesis in the developing dermamyotome and is suggested to control the expression of genes that guide lateral migration of muscle precursors and maintain their migratory potential.^78^ Lbx1 is also critical for specification of dorsal interneuron fates and somatosensory function.^79^ Thus, *LBX1* may be implicated in myogenic and neurogenic etiology of human AIS.

## Genetics of AIS curve progression

Identifying patients with a high likelihood of curve progression is a top priority of AIS clinical management. Consequently, a comprehensive search for severity-correlated SNPs was conducted in a GWAS by limiting cases to those with spinal deformity above 40 degrees after skeletal maturity or surgical correction. Through a two-stage association study using a total of 12 000 Japanese subjects, a novel locus, rs12946942 on chromosome 17q24.3, showed significant association and was replicated in a Han Chinese population.^80^ rs12946942 and the LD block containing it are in a gene desert between *SOX9* and *KCNJ2*. The block contains six enhancer elements, one of which forms a long-range chromatin loop to *SOX9* in a prostate cancer cell line, and SNPs within the enhancer appear to direct allele-specific gene expression. It is posited that variants in the region may confer significant risk of progressive AIS by controlling scoliosis-related spatio-temporal *SOX9* expression.

A later study in the Japanese cohort identified association with SNPs in MIR4300HG, the gene encoding MIR4300 microRNA. Correlated SNPs were mapped within a putative enhancer in a conserved toplogically associated domain (TAD). So-called TADs are large, self-interacting genomic regions that are implicated in gene regulation.^81^ Although MIR4300 expression has been noted in bone, cartilage, IVD, and spinal cord among other tissues, its function and potential role in AIS is not yet known.^82^

## The IVD matrisome and rare genetic variation

Heritability studies (calculated as the square of the correlation coefficient, *r*^2^) estimate that <7% of the total genetic variance underlying AIS has been discovered.^67^ More recently, DNA sequence-based approaches have been applied to both AIS case/control cohorts and to individual AIS families, with the goal of identifying rare disease-causing variants that would be missed by standard GWAS and that could account for “missing” heritability.^83^ The discovery of rare disease alleles has been bolstered by large-scale publicly available reference sets such as the Genome Aggregated Database (gNOMAD) that provide summary data for sequenced alleles.^84^ The search space for rare genetic risk factors is often limited to the exons of the genome, the so-called “exome” for practical reasons, e.g., cost, computation time, and biologic interpretability. As the costs of sequencing decrease, the field is moving beyond exome sequencing (ES) toward genome sequencing (GS). Simultaneously, the tools for identifying and interpreting noncoding rare disease variants are rapidly improving.^85^ Single rare variants may be tested for enrichment in cases versus controls or co-segregation with disease in families. Alternatively, a potentially more informative approach is to statistically measure the burden of rare alleles per gene (i.e., coding sequences) in cases versus controls. The first ES burden study of 91 AIS cases and 337 controls showed *FBN1* and *FBN2,* encoding fibrillins-1 and -2, respectively, as the most significantly associated genes. Dominantly inherited mutations in *FBN1* are known to cause Marfan syndrome (OMIM 154700). Marfan syndrome patients typically present with distinctive skeletal features including increased height, disproportionately long limbs and digits, anterior chest deformity, mild-to-moderate joint laxity, scoliosis, dural ectasia (widening of the dural sac around the spinal cord), and a narrow, highly arched palate with crowding of the teeth. Cardiovascular features can be life-threatening and include mitral valve prolapse, mitral regurgitation, dilatation of the aortic root, and aortic regurgitation.^86^ Mutations in *FBN2* are associated with the related disorder Congenital Contractural Arachnodactyly (CCA) (OMIM 121050). The mutations identified in AIS tended to be associated with tall stature and larger spinal curves, but without the cardiac manifestations or other complications that are typical of MFS or CCA,^87^ suggesting they exert hypomorphic effects on the functions of these proteins.

Other sequence-based studies have highlighted rare mutations in ECM genes. In a pathway-based analysis of severe (i.e. >40 degree curves) AIS, restricting the analysis to novel (not found in dbSNP at that time) coding variants revealed association with musculoskeletal collagen genes, particularly *COL11A2*.^88^ Dominant inheritance of mutations in *COL11A1*, *COL11A2*, and *COL2A1* cause Stickler or Marshall syndromes (OMIM 604841, 184840, 154780). The Stickler/Marshall syndromes are hereditary conditions characterized by distinctive, flattened facial features, abnormalities of the vitreous, conductive hearing loss, scoliosis or kyphosis, and joint problems including joint hypermobility and early onset arthritis. Many Stickler patients exhibit Pierre Robin sequence, a combination of features (cleft palate, glossoptosis, micrognathia) that can lead to feeding problems and difficulty breathing.^89,90^ On average, significantly more novel variants in ECM genes were observed in the AIS cohort compared with controls, suggesting that the polygenic accumulation of novel variants within potentially many ECM genes contributes to AIS risk. More recently, an exome-wide association study that was restricted to severe AIS identified a variant in the *SLC39A8* gene. *SLC39A8* is a ubiquitously expressed divalent metal cation transporter that, not surprisingly, has been genetically linked to disorders of numerous organ systems in humans including blood pressure, body mass index (BMI), circulating manganese levels, and circulating cholesterol levels.^91^ The AIS-associated variant altered alanine at position 391 to threonine. This single amino acid change was also correlated with greater spinal curvature, decreased height, increased BMI, and lower plasma manganese in AIS cases. Mutations in the zebrafish homolog caused vertebral anomalies and mild spinal curvatures that were noted to be reminiscent of zebrafish knockouts of the gene encoding alpha-1 chain of type VIII collagen described below.^92^ This observation suggests the possibility of a functional interaction between *Slc39a8* and the extracellular matrix in zebrafish, and possibly other vertebrates including humans. Taken together, rare variant-based studies are interpreted to suggest that rare, hypomorphic variants in genes responsible for Mendelian connective tissue disorders may be contributing factors in AIS.

Families with multiple affected members provide the opportunity to map and identify disease variants by their co-segregation with AIS. In one study, filtering for rare variants (minor allele frequency <5% in the Exome Aggregation Consortium (EXAC)) identified by ES in five multigenerational families followed by gene ontology term enrichment analyses produced top results for actin- and microtubule-based projections and ECM.^93^ In a separate hypothesis-driven study, the *HSPG2* gene encoding the SLRP perlecan was selected as a candidate per ES of an extended AIS family. One particular *HSPG2* variant, Asn786Ser, was marginally overrepresented in a larger case-control cohort.^94^ Recessively inherited mutations in the *HSPG2* gene cause Schwartz–Jampel syndrome type I (OMIM 255800), a rare disorder characterized by joint contractures, myotonic myopathy (muscle stiffness), dystrophy of epiphyseal cartilages, eye abnormalities, short stature, distinct facial features and propensity to malignant hyperthermia.^95^ In a separate study, linkage analysis and ES of a Swedish pedigree with an autosomal dominant inheritance of AIS identified a rare, nonsynonymous variant (V2287I) in the *CELSR2* gene encoding cadherin EGF LAG seven-pass G-type receptor 2. Furthermore, analysis of tagging SNPs revealed significant association (*P* = 0.000 1) to this variant in a set of 1 739 Swedish-Danish AIS cases and 1 812 controls but not with case-control cohorts from Japan and the US. It is interesting to note that *CELSR2* is a planar cell polarity cadherin, like *GPR126*, that encodes an adhesion-class G protein receptor. Mice that lack *Celsr2* develop progressive hydrocephalus and display dysgenesis of ependymal cilia. Further family-based studies hold promise for discovering higher-impact AIS disease alleles, although reduced penetrance of the phenotype is an acknowledged challenge. Continued genome sequencing in both populations and families is warranted to fully define the polygenic architecture of the disease.

## Models of AIS pathogenesis

Taken together, evidence from human genetic studies and animal models supports a role for vertebral cartilages, particularly the cartilage matrisome, in AIS pathogenesis. This model focuses attention on the IVD due to its role in withstanding and dissipating the forces of spinal growth. Radiographic studies of the spine in patients with progressive scoliosis are consistent with this model. In AIS patients that were followed longitudinally, wedging of the involved discs preceded wedging of the involved vertebrae until the end of the growth spurt, at which point vertebral wedging progressed somewhat more.^96^ Furthermore, MRI studies of AIS patients found that progressive scoliosis was associated with displacement of the NP toward the convexity of the curve.^96^ Whether the relatively subtle hypomorphic genetic changes in components of the IVD ECM identified with AIS so far are necessary or sufficient to cause AIS is not clear at present. Consequently, the ability to affect spatio-temporal expression of specific genes with precision, and to create knock-ins of disease-associated mutations in vertebrate models will be key for addressing these questions.

The specific tissues in which AIS originates has been difficult to discern, as bone and soft tissue components of the spine in human patients appear to develop and function normally, at least superficially. Likewise, although AIS manifests around the adolescent growth spurt, it has been unclear whether its origins trace back to deficiencies in early development. Research addressing these questions has been severely limited by a lack of appropriate animal models that recapitulate a true AIS phenotype. This issue is likely a reflection of several factors, including reduced penetrance and the relatively late onset that may be missed in typical forward genetic screens. Moreover, the genetic factors identified in humans thus far point to hypomorphic mutations and variations in regulatory elements that may manifest in tissue- and temporal-specific ways. Fortunately, breakthroughs in this regard have come from use of the Cre/loxP system to create tissue-specific and inducible knockouts of AIS candidate genes. For example, removing *Gpr126* using a Col2Cre driver produced thoracic scoliosis beginning at age P20 and becoming more prevalent with age. From this study it was posited that AIS may arise from deficiencies in a specific osteoprogenitor cell type expressing type II collagen.^97^ The study also suggested that scoliosis may have its beginnings in early development. While cell proliferation was unaffected in rib and spinal cartilage (IVD) cells, a slight increase in apoptotic cells was noted in both locations at P1 before the expected onset of spinal curvature. This and additional experiments defined a critical role for *Gpr126* on cartilage development, including effects on the morphogenesis of the annulus fibrosis, chondrocyte survival, and the expression of *Gal3st4* encoding galactose-3-O-sulfotranserase 4.^98^ These results also support the hypothesis that the dysregulation, and possibly loss, of specific cell populations early in postnatal development leads to a “weakened” IVD, perhaps through deficiencies in the ECM, that is vulnerable to the increasing axial and torsional forces exerted during rapid growth.

Fish (teleosts) are emerging as powerful tools for understanding developmental mechanisms of spinal deformities. Scoliotic curves have been observed in various fish species, including early studies in “curveback” guppy that demonstrated a heritable phenotype reminiscent of human idiopathic scoliosis.^99^ It has been conjectured that the cranial to caudal spinal loads that fish experience while swimming may recapitulate, to some extent, the same directional forces on the human spine due to walking upright.^100^ Zebrafish (*Danio rerio*) in particular is a workhorse laboratory fish that has become a staple in the armamentarium of tools to dissect human developmental disorders. Attractive features of the zebrafish include its relatively rapid generation time of 6–12 weeks, external and near-transparent development, and genetic and genomic tractability. Early models of spinal deformity in zebrafish were mapped to mutations in the *Col8a1a* gene encoding alpha-1 chain of collagen type VIII. These mutants exhibited vertebral anomalies, reminiscent of human “congenital” scoliosis, that were correlated with early defects of notochord development.^101^ Similar morphologic changes were later noted in *Slc39a8* knockout zebrafish as described above, raising the question of a possible pathogenic overlap between congenital scoliosis that involves frank vertebral anomalies, and idiopathic scoliosis in which the vertebrae appear to be morphologically normal. Evidence in support of this came from another gene-targeting study in zebrafish that produced the first genetically defined developmental model of IS. In that study, genetic removal of an atypical protein tyrosine kinase called *Ptk7* evoked a rotational spinal deformity beginning at late larval stages and progressing until sexual maturity. This model also recapitulates human IS in that there was a strong bias toward severe curves in females. Further genetic manipulations that removed maternal copies of *Ptk7* in early development led to loss-of-function mutants with vertebral anomalies reminiscent of congenital scoliosis, suggesting that the same genetic mutations could result in idiopathic versus congenital scoliosis phenotypes in a context-dependent manner.^102^ This study of *ptk7* mutants implicated abnormal spinal fluid flow. A more recent study further implicated neuroinflammation as an underlying cause of spine curve formation in *ptk7* mutants.^103^ A correlation between inflammation and onset of scoliosis was also supported by independent studies in which the transcription factor Stat3 was removed from maternal and zygotic zebrafish. During later development, *stat3* mutant zebrafish exhibit stunted growth, scoliosis, excessive inflammation, and fail to thrive. In humans, dominant-negative mutations in *STAT3* cause Hyper IgE syndrome marked by dysregulation of immunity as well as late-onset scoliosis reminiscent of AIS.^104^

The ability to create knock-ins of specific human mutations, whether in exons or in disease-associated enhancers such as *Xe1/PEC7*, using genome editing tools will be critical for hypothesis-testing in vertebrate systems. It remains to be seen whether single variants in individual genes will be sufficient to create AIS-like phenotypes in mouse. More likely, in keeping with the complex heritability of AIS, it may be combinations of genetic changes affecting multiple components of the cartilage matrisome that will be required to evoke progressive deformity. The introduction of multiple genetic changes into individual animals by breeding strategies or sequential genome editing may be required to model AIS appropriately.

## Therapeutic strategies for AIS

Natural-history studies have shown AIS measuring less than 30 degrees at the cessation of growth rarely increases in adulthood. Fortunately, the majority of patients are in this category but nevertheless require careful monitoring with radiography to monitor the curve. Curves greater than 50 degrees, however, will slowly progress throughout adulthood at a rate of 0.75–1.00 degrees per year. Therefore, the AIS patient with an immature skeleton and scoliosis of greater than about 20 degrees is at risk for progression and may be treated by bracing.^14^ Bracing addresses both biologic and biomechanical susceptibility in AIS by relieving asymmetric loading in the spine and thereby allowing symmetric growth. Although bracing has shown efficacy in preventing progression in clinical trials,^8^ it is typically prescribed to be worn 12–16 h each day over a course of years, and compliance is notoriously poor.^8^^,105^ Surgical correction is required when bracing fails to halt progression, not only to address the appearance of the deformity but also to prevent future pulmonary complications. Lung volume normally increases dramatically during the juvenile and adolescent years, approximately doubling from the age of 10 years to skeletal maturity. Progressive AIS is associated with diminished lung volumes, particularly for curves ≥70 degrees. Patients with curves reaching 100 degrees are at significant risk of symptomatic restrictive pulmonary disease. Given all of these issues, the first goal of surgical approaches to AIS is to halt progression beyond an acceptable limit of ~50 degrees Cobb angle, while a secondary goal is to achieve some correction of the existing deformity.^1^

The fact that bracing can be successful suggests that future biologic therapies aimed at preventing progression should target the same “window of opportunity”. As outlined in this review, genomic studies of human AIS point to the biogenesis and/or maintenance of the ECM of the IVD in disease susceptibility. Future therapies consequently could be aimed at stimulating proper ECM production in cells of the IVD in patients with mild AIS, with the goal of providing better biomechanical and/or biologic resistance to asymmetric growth forces. Approaches to IVD-targeted therapy could perhaps build upon the data armamentarium developed by the intervertebral disc degeneration (IVDD) research community, as well as on other successful gene therapy efforts.^106–109^ Therapies proposed to stimulate ECM production in IVDD include local delivery of growth factors or viral-based gene therapy, or adoptive transfer of adult mesenchymal stem cells. In this regard, the avascular nature of the IVD and its limited systemic connection could prove advantageous for direct in vivo therapeutic delivery, similar to the eye. Direct injection of therapeutic viral vectors has shown such promise for correcting several inherited eye disorders and it is now being pursued in clinical trials.^108^ In IVDD, increased proteoglycan synthesis has been observed after direct injection of adenovirus, adeno-associated virus, or lentivirus expressing *Tgf-β*, latent membrane protein 1 (*Lmp1*), *Sox9*, and other factors in rabbit models.^106^ Cell-based therapies would aim to replace or regenerate resident IVD cells, likely using endogenous progenitor cells or MSCs to increase ECM production. However, harvesting endogenous cells would require surgery, and the cells would be predicted to harbor the same genetic susceptibilities as resident IVD cells. The number of resident NP cells in the IVD is expected to be at its peak in individuals younger than 20 years of age.^107^ Although the cellular status in AIS patients is not well understood, delivering additional progenitors into the IVD environment might not prove beneficial. Modern genome editing technologies provide another promising line of gene therapy. Specifically, clustered regularly interspaced short palindromic repeat (CRISPR)-associated nucleases can be used to cleave specific sites, with subsequent removal or replacement of targeted DNA sequences.^108^ The benefits of genome editing are its simplicity and precision in removing or correcting an offending mutation. This is particularly relevant where simply stimulating endogenous gene expression would not apply. For example, genome editing could be envisioned to correct aberrant amino changes in type XI collagen in the AIS IVD. Another advantage of CRISPR-based genome editing is the efficiency of creating multiple genetic changes within each targeted cell, which may be necessary for more complex disorders such as AIS. It should be noted that while genome editing holds promise, its clinical application to any acquired or inherited human disorder is in its infancy until potential side effects (off-target changes and possible leakage to germline) are fully understood and controlled. Considering AIS, biologic therapies are particularly attractive given the “window of opportunity” in children with mild AIS who are apparently otherwise healthy.

Prevention of AIS altogether has been a futuristic goal but not inconceivable given the emerging understanding of underlying mechanisms. For some patients, improving mechanical function in the IVD by boosting glycoprotein content and hydration early in life could be envisioned as a preventative strategy, perhaps by small molecule or peptide therapies, or by better nutritional delivery to the disc.^110^ In this scenario, the late onset and ostensibly healthy metabolism in AIS could prove advantageous, analogous to preventing osteoporosis by early calcium and vitamin D intake. It is notable however that in the IVD, the ECM is key in governing the rate of molecular transport through the long pathway from the cartilaginous endplate to the relatively sparse NP and/or AF cells. Aggrecan in particular intercalates with the collagen framework and thereby largely determines solute distribution and mobility.^110^ Clearly, appropriate in vitro and in vivo model systems and more fundamental understanding of the genetic, cellular, and developmental mechanisms of AIS are needed to advance any biologic therapeutic strategies.

## Summary

The embryologic development of the spine is an exquisitely controlled process both spatially and temporally. Deleterious mutations in single genes controlling somitogenesis can lead to rare Mendelian disorders affecting spinal integrity. Idiopathic scoliosis, usually occurring at adolescence, is the most common developmental disorder of the spine in children. AIS is genetically complex, but emerging data both from GWAS and next-generation sequencing of human AIS cohorts have highlighted a role for determinants of cartilage biogenesis and development of the IVD, particularly pathways controlling extracellular matrix, or matrisome production. DNA sequence variants have been identified that potentially alter the transcriptional regulation of matrisome genes, or that create hypomorphic changes in protein-coding sequences. Combinations of these variations may effectively create tissue-specific alterations, with major effects on the composition and/or organization of the IVD matrisome in cells of both NP and AF. Cellular maturation in the NP occurs in the first decade of life in humans and also may be a contributor to AIS. In either scenario, impairments in the disc may cause asymmetric responses to the strong torsional forces along the spine during adolescent growth, leading the spine to twist away from these forces. In contrast to the disc in adult IVD degeneration, the IVD in AIS does not exhibit overt signs of degeneration. Given this and the fact that current bracing therapies during the adolescent growth spurt can be effective suggests that there is a window of opportunity for providing biologic therapies to control or prevent AIS and subsequent curve progression. The IVD of AIS patients may be amenable to direct in vivo delivery of molecular or nutritional therapies aimed at preserving its structural integrity. These factors encourage optimism and justify continued research to understand underlying disease mechanisms and to develop robust model systems for in vivo testing.
